# PROTOCOL: Interventions to Reduce or Prevent the Effects of Climate Change on Populations Vulnerable to Poverty and Social Exclusion in Low- and Middle-Income Countries: An Evidence and Gap Map

**DOI:** 10.1177/18911803261461576

**Published:** 2026-07-21

**Authors:** Alfonso Apicella, Sandy Oliver, Janice Tripney

**Affiliations:** 1 EPPI Centre, Social Science Research Unit, Social Research Institute, University College London, London, UK

**Keywords:** poverty, social protection, vulnerable populations, well-being, social exclusion, low- and middle-income countries, climate change, climate adaptation response, global warming, evidence and gap map, systematic review, process evaluation, Indigenous and local knowledge

## Abstract

This is the protocol for a Campbell evidence and gap map (EGM). The objectives are as follows: (1) to develop a systematic taxonomy of intervention types and well-being outcomes to organise the evidence on climate-related interventions for populations vulnerable to poverty and social exclusion in low- and middle-income countries (LMICs); (2) to map existing systematic reviews to show what we know (evidence) and do not know (gaps) about the effectiveness of interventions to reduce or prevent the effects of climate change on these populations, with systematic reviews of process evaluations mapped alongside effectiveness reviews to illuminate the mechanisms, barriers and facilitators that shape intervention outcomes; (3) to provide structured database entries for included reviews, summarising the intervention, context, study design, sources of evidence and main findings, and appraising each review’s methods using a standardised checklist; and (4) to provide a searchable database that maps relevant systematic reviews by intervention type and outcome category to support policy decision-making.

## Background

### Introduction

#### The Problem, Condition or Issue

Climate change stands as one of the most pressing global health threats of the twenty-first century (World Health Organisation, 2023). Its far-reaching social ramifications, which permeate multiple dimensions of human well-being, are profound and manifest in inequitable patterns (Mearns & Norton, 2010).

The concept of ‘planetary boundaries’, introduced by Rockström et al. (2009), delineates nine critical environmental thresholds that define the safe operating space for humanity, essential for preserving not only the stability and resilience of the Earth system but also the social and economic structures that depend upon it. Within this framework, climate change denotes disruptions in global climate patterns driven by an energy imbalance resulting from escalating greenhouse gas concentrations (Richardson et al., 2023). These disruptions not only drive rising global temperatures but also in turn exacerbate social inequalities, threaten livelihoods and undermine human well-being worldwide, with disproportionate impacts on resource-poor communities (IPCC, 2023; Romanello et al., 2024).

The World Meteorological Organisation (WMO) recently stated that the year 2024 was the warmest on record and marked a decade of unprecedented heat caused by human activities (WMO, 2025). In 2023, people were exposed to, on average, an unprecedented 50 more days of health-threatening temperatures than expected without climate change. In addition, extreme drought affected 48% of the global land area – the second highest level ever recorded – and the higher frequency of heatwaves and droughts was associated with 151 million more people experiencing moderate or severe food insecurity annually between 1981 and 2010 (Romanello et al., 2024).

In a similar vein, over the past two decades, climate-related heat mortality has risen by an estimated 54%, attributed to the effects of climate change, particularly among older populations with pre-existing health conditions (Watts et al., 2021). Older adults are especially susceptible to heat-related illnesses, while infants under one year are at heightened risk from heat exposure and dehydration (Romanello et al., 2021). From a psychological standpoint, Miles-Novelo and Anderson (2022) argue that the changing climate will result in increasing conflict prevalence.

Nowhere is this more apparent than amongst individuals and communities in low- and middle-income countries (LMICs) who bear a disproportionate burden of climate change, compounded by pre-existing social vulnerabilities, including limited adaptive capacity and inadequate social protection systems (Hvinden & Schoyen, 2022). Climate change has intensified multiple, interlocking vulnerabilities to poverty and social exclusion in LMICs, disproportionately harming populations with the least adaptive capacity. Recent studies document worsening food and nutrition insecurity as rising temperatures engender malnutrition and stunting (Bianco et al., 2024; Fanzo et al., 2025). Other relevant research links climate shocks to deteriorating physical and mental health, increased disease burdens and heightened economic precarity, particularly amongst rural and coastal communities that rely on climate-sensitive livelihoods (Moore & Niles, 2025; Naser et al., 2024). Furthermore, research also points to how recurring climate shocks can entrench poverty and inequality by eroding household assets, straining public systems and slowing broader development gains in LMICs (Dang et al., 2025).

Against this backdrop, the financing decisions of governments and international institutions – including continued subsidies for fossil fuels – reflect political economy dynamics that shape both the drivers of climate change and the design of interventions to address its consequences. Financing for development itself has been severely weakened, particularly from early 2025 onwards (OECD, 2026). A major turning point came in February 2025, when USAID – the world’s largest provider of international development aid – was shut down by the US government, resulting in the termination of thousands of international development and humanitarian programmes (Gordon, 2025). At the same time, several European donor countries, including the UK, France, Germany, Belgium and the Netherlands announced further reductions in their aid budgets. Taken together, these decisions represent a broader retreat from international development financing and substantially limit the already constrained resources available for achieving the Sustainable Development Goals, particularly in low- and middle-income countries (Gordon, 2025). Moreover, in April 2026 the United Nations and the Organisation for Economic Co-operation and Development (OECD) almost simultaneously released reports on development finance, revealing data on official development assistance (ODA) for 2025 that point to a dramatic 23% decline in a single year – the largest drop the world has ever seen. This sudden halt affected key areas such as health, food assistance and climate adaptation, with the harshest consequences hitting the most vulnerable populations at risk of poverty and social exclusion. The financing of interventions to reduce or prevent the effects of climate change on populations vulnerable to poverty and social exclusion in LMICs is at stake vis-à-vis a growing development financing gap.

In this context, there is a clear need to strengthen the evidence base informing development and climate finance and decisions. To support decisionmakers and researchers, this evidence and gap map (EGM) seeks to systematically identify, organise and synthesise existing evidence on interventions to prevent or reduce the effects of climate change on populations vulnerable to poverty and social exclusion in LMICs.

The EGM is designed to capture a wide range of intervention types, financing mechanisms and sources of evidence and knowledge used, including Indigenous and local knowledge alongside social research evidence. In doing so, it equips subsequent research to examine how different types of knowledge are generated, selected and used in development finance decisions affecting the populations under study.

#### The Intervention

This EGM will adopt Bronfenbrenner’s (1979) socio-ecological model as a framework for understanding eligible interventions. A recent study by Xue et al. (2024) provides a pertinent example of this model’s application in mapping mental health and psychosocial interventions in the context of climate change, demonstrating the model’s capacity for comprehensive analysis. This model will allow for the categorisation of interventions across multiple, intersecting levels to enable a nuanced understanding of the following levels:

• **Micro-system level:** Interventions targeting individuals and households, such as mental health support or family-based initiatives, as well as interventions within health facilities focusing on individuals and households.
• **Meso-system level:** Interventions strengthening, for example, healthcare services or farming interventions aimed at specific groups or communities.
• **Exo-system level:** Interventions that influence broader systems, including policy changes designed to improve access to healthcare or economic support. These may include financial sector or legal innovations impacting entire constituencies.
• **Macro-system level:** Interventions addressing cultural, economic and political factors, including national policies and public awareness campaigns. These might cover areas such as hygiene or education policies.

To deepen the analysis and enhance the categorisation of interventions, the study also utilises the World Bank's (2025) sector taxonomy, also adopted by the International Initiative for Impact Evaluation (3ie). This well-established taxonomy is accessible through 3ie’s *taxonomy explorer*. It furnishes a solid, pre-existing tool for identifying relevant international development interventions.

#### Why It Is Important to Develop the EGM

No EGMs are known to exist or be underway on this topic.

## Objectives

The EGM presents studies on interventions to reduce or prevent the effects of climate change on populations vulnerable to poverty and social exclusion, mapped across a range of well-being outcome domains.

Specifically, the objectives of the EGM are to:

(a) Develop a systematic taxonomy of intervention types and well-being outcomes to organise the evidence on climate-related interventions for vulnerable populations.
(b) Map existing systematic reviews to show what we know (evidence) and do not know (gaps) about the effectiveness of interventions to reduce or prevent climate change impacts on populations vulnerable to poverty and social exclusion in LMICs. Systematic reviews of process evaluations are also mapped alongside effectiveness reviews as they can illuminate the mechanisms, barriers and facilitators that shape intervention outcomes, including through qualitative and views-based evidence.
(c) Provide structured database entries of included reviews, summarising the intervention/s, context (setting/population), study design, sources of evidence used and main findings, and appraising each review’s methods using a standardised checklist.
(d) Provide a searchable database that maps relevant systematic reviews organised by intervention type and outcome categories, accessible to users through 3ie’s online tool, to support and inform policy decision-making.

## Methods

### Evidence and Gap Map: Definition and Purpose

Evidence and gap maps (EGMs) are visual tools that systematically present and organise existing evidence on specific topics, particularly in policy-relevant domains. They are designed to provide a structured overview of the available research, including areas where evidence is clustered and where critical gaps exist, making them especially valuable for translating large volumes of research into an accessible format that can inform decision-making in research, policy and practice. EGMs are especially valuable for summarising large volumes of research evidence in an accessible, user-friendly format.

This EGM adheres to the standards established by White et al. (2020) and draws on the methodological framework outlined by Snilstveit et al. (2016). This EGM is specially designed to map evidence, or the lack thereof, within the thematic area of international/sustainable development and climate change. It will identify completed and ongoing systematic reviews that assess the effectiveness of eligible interventions, as well as reviews that examine process evaluations or other studies of barriers and facilitators affecting implementation.

**Figure 1.**
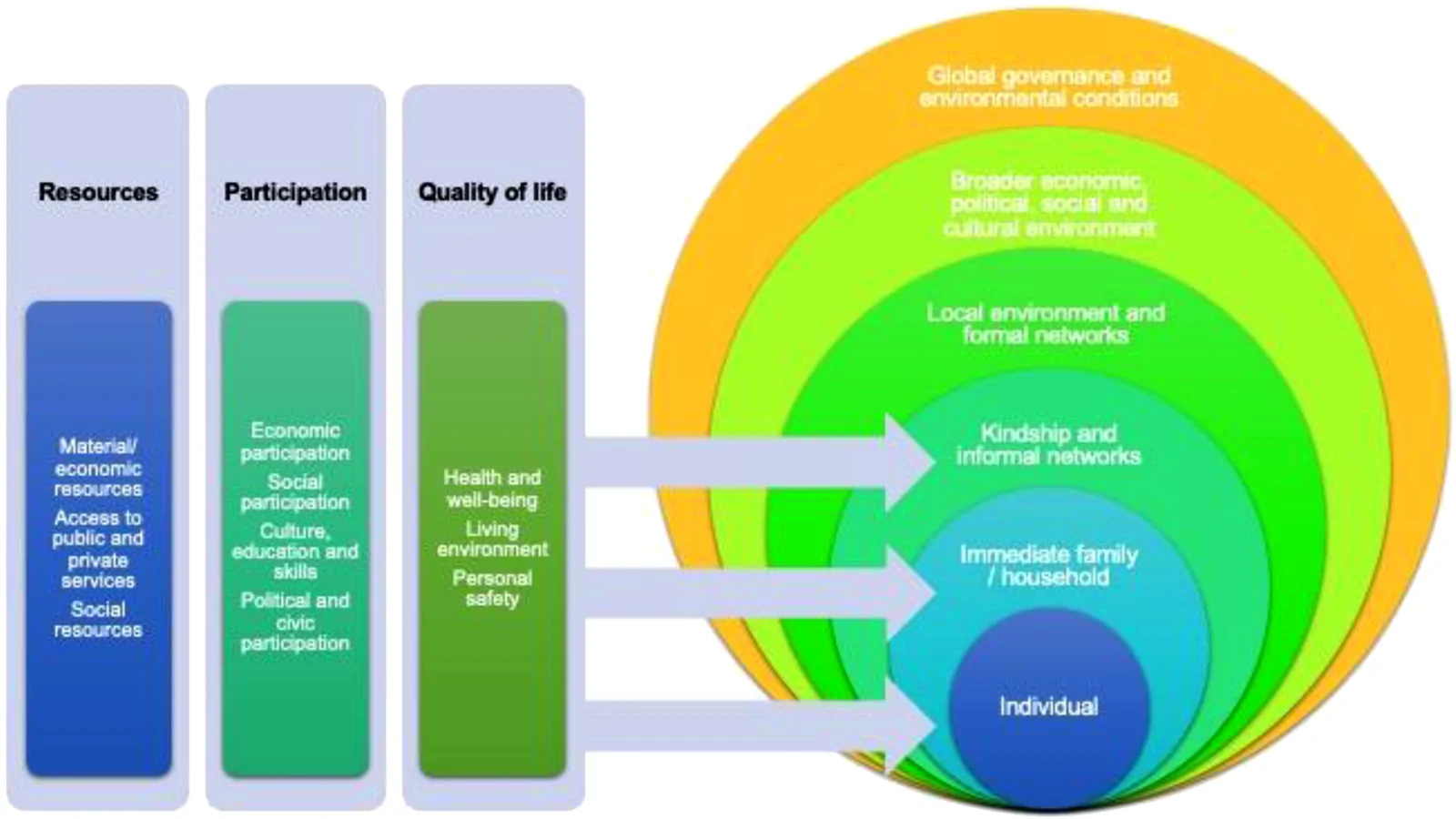
Conceptual Framework. Source: Own elaboration based on Levitas et al.’s (2007) and Bronfenbrenner’s (1979)

The included reviews will be mapped onto the matrix in Table 1 below, using a bespoke integral well-being framework, as shown in Figure 1 below. The results will be presented visually to represent the volume of evidence for different exposure-outcome combinations. Rather than synthesising findings across contexts, the EGM will characterise the evidence landscape, including localities, demographic groups, sources of evidence, etc., thus making visible both where synthesised evidence clusters and where contextual gaps remain.

**Table 1.** Intervention-Outcome Framework as Dimensions of the Evidence and Gap Map (EGM)

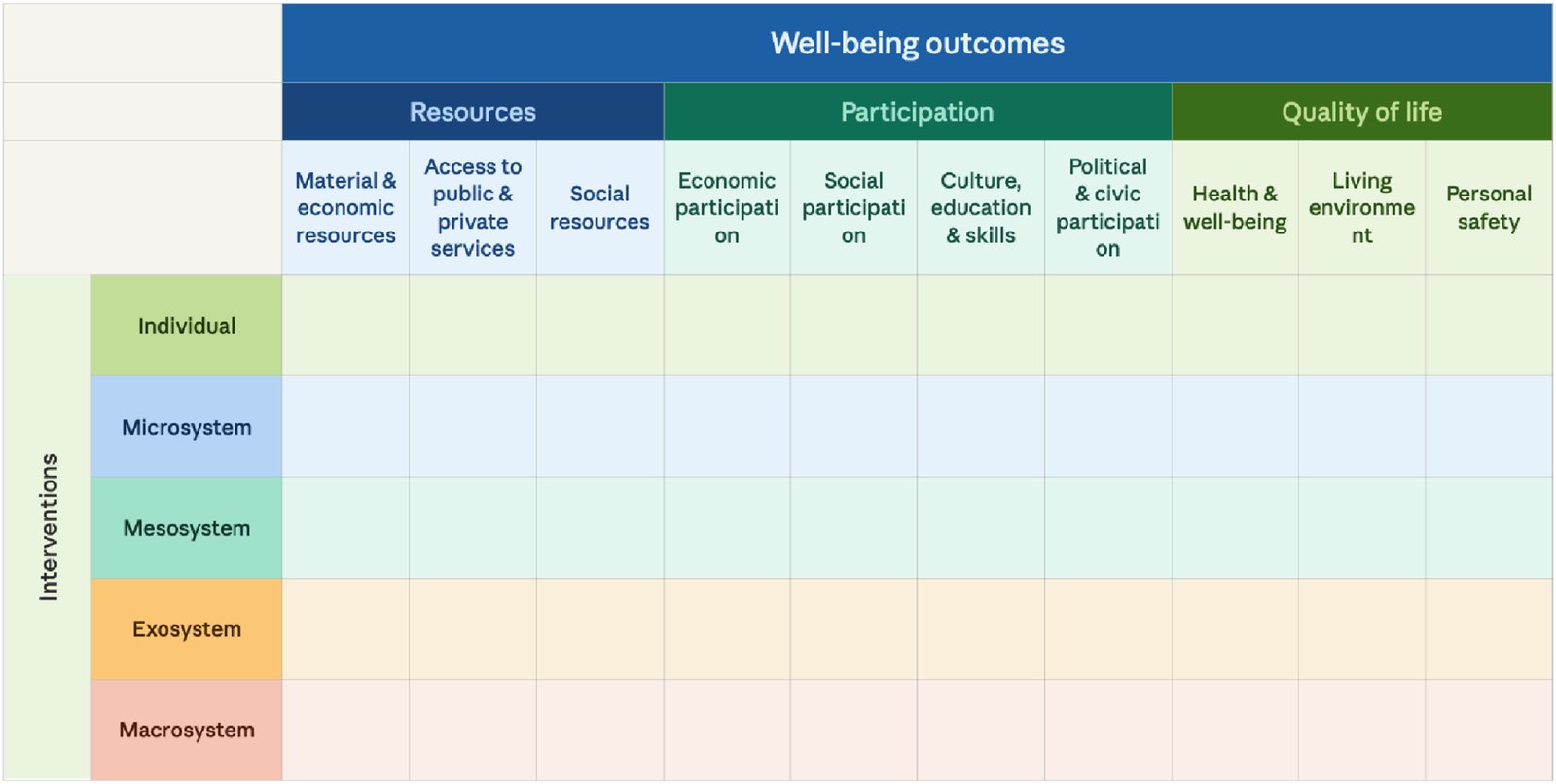

*Note.* Adapted from the author’s own analysis, based on Levitas et al. (2007) and Bronfenbrenner (1979).

This approach acknowledges that systematic reviews, as the unit of analysis, tend to generalise across contexts. In order to handle heterogeneity, information about different geographies, populations and sources of knowledge used will be treated as a substantive, mappable finding via specific filters. A confidence rating will be used to assess the quality of the reviews. The resulting EGM will be made available through EPPI Mapper software, as an online interactive platform which creates a map of review data to facilitate the dissemination of findings.

In addition to the map, a detailed EGM report will be produced, which will include an analysis of the characteristics of the available evidence and key trends, such as the number of reviews published over the selected timeframe, geographical focus, emphasis on exposures and outcomes, types of knowledge used and the intended target audiences of the interventions.

The strategy underpinning this EGM will allow for the inclusion of additional studies identified through iterative searching, given the fast-paced nature of this area of investigation, ensuring that new data and developments are integrated into the analysis. As new reviews become available, they will be assessed for their relevance to the study and incorporated into the EGM if they meet the established eligibility criteria. Lastly, the research programme aimed at evaluating user needs and practices for climate and health evidence synthesis, currently co-run by the UCL EPPI Centre, will be considered for applications of automation tools for evidence surveillance in the field of climate and health (Bond et al., 2024), as it falls within the scope of this study.

### Framework Development and Scope

The framework for this EGM is being developed through a systematic and consultative process involving multiple stakeholders through an Advisory Group, alongside reference to existing theoretical frameworks. This process ensures that the EGM is both substantively and methodologically rigorous, context-sensitive and policy-relevant. The steps include:

• Consultation with stakeholders:
  ◦ An Advisory Group has been established, comprising academics, policymakers, practitioners and independent researchers. The diversity of expertise ensures the framework aligns with real-world needs and enhances its policy relevance.
  ◦ Advisory Group members have provided feedback at the beginning of framework development and will be consulted at key stages of the EGM to ensure that the framework remains comprehensive, context-sensitive and aligned with the needs of users in research, policy and practice.
  ◦ The framework will be refined based on this feedback, ensuring it remains responsive to emerging needs and challenges.
  ◦ Stakeholders will play a key role in informing the overall conceptual framework and reviewing it as it evolves, together with the EGM authors. Meetings will be planned throughout the development phase to gather feedback and refine the approach, as outlined in the Terms of Reference document (Appendix A).
• Engagement with policy and strategic documents:
  ◦ This EGM will draw on key international policy documents, surveys and studies, such as the Poverty and Social Exclusion portal (https://www.poverty.ac.uk) of the University of Bristol as a framework for measuring poverty and informing policy using the consensual approach. This framework informs the design of the intervention-outcome matrix.
• Integration of existing theoretical frameworks:
  Bronfenbrenner’s (1979) socio-ecological model will be adopted to categorise interventions at multiple levels (e.g., micro-, meso-, exo-, macro- and chronosystems). This layered approach allows for a comprehensive understanding of the interplay between individual, societal and systemic factors.
  ◦ The aforementioned model is adopted alongside the taxonomy of interventions developed by the World Bank (2025) and currently used by 3ie (International Initiative for Impact Evaluation) for more granular classifications within each level. This taxonomy provides a structured classification of intervention types, such as financial incentives, service delivery reforms and regulatory changes, ultimately allowing for a more precise categorisation of interventions.

In terms of scope, this EGM will include two types of systematic reviews: those assessing the effectiveness of interventions and those examining process evaluations, as they capture not only what works but also the mechanisms, barriers and facilitators that shape well-being outcomes, including through qualitative and views-based evidence. Both types of reviews concern interventions to reduce or prevent the effects of climate change on populations vulnerable to poverty and social exclusion in LMICs, with a particular focus on the following key aspects:

(1) **Populations:** The EGM will focus on individuals, households and communities in LMICs who are vulnerable to poverty and social exclusion. This will be operationalised using the Bristol Social Exclusion Matrix or B-SEM (Levitas et al., 2007), which provides a structured, multi-dimensional framework across 10 domains organised under three fields: resources (material and economic resources; access to public and private services; social resources); participation (economic participation, social participation, culture, education and skills and political and civic participation); and quality of life (health and well-being, living environment and crime, harm and criminalisation). The B-SEM derives from the consensual approach to poverty and deprivation measurement pioneered by Mack and Lansley (1985), whose underlying rationale, grounding categories of poverty and social exclusion in socially negotiated rather than expert-imposed criteria, makes it transferable across different contexts, including in LMICs.
(2) **Interventions:** The EGM will include different types of intervention, which will be catalogued using a robust framework that organises interventions by section, including but not necessarily limited to:
  (a) Social and behavioural interventions (e.g., community-based education, mental health support).
  (b) Technological innovations (e.g., climate-resilient crops, early warning systems).
  (c) Public and social policy measures (e.g., urban planning, social protection schemes).
  (d) Health, environmental and capacity-building interventions.
(3) **Outcomes:** The EGM will map interventions against resources, participation and quality of life.
(4) **Framework dimensions:** The primary dimensions of the EGM will include intervention types and well-being outcomes, organised according to the poverty and social exclusion dimension of the Levitas et al.’s (2007) framework. In addition, further filters such as study design, geographic location and sources of knowledge used will be incorporated.
(5) **Geographic and contextual focus:** The EGM will explore the available evidence in LMICs, with particular emphasis on rural, urban and climate-vulnerable regions, including Small Island Developing States (SIDS) and arid zones. This contextual focus will ensure that the EGM reflects the unique challenges faced by these regions, which are disproportionately affected by climate change.

The framework developed through this process will inform the inclusion and exclusion criteria of the EGM, outlined below, ensuring that the map remains focused on relevant evidence that can support decision-making in policy, practice and research.

### Stakeholder Engagement

Stakeholder engagement will be integral to the development of this EGM to ensure its relevance and practical usability for end users. In this process, stakeholders will discuss their various perspectives and so contribute to refining the research questions and processes. This will ensure that the EGM is aligned with real-world and academic challenges, thereby making it a usable resource for policymakers, philanthropic foundations and researchers.

In order to facilitate their engagement, a formal Advisory Group was convened in 2024, bringing together professionals from academia, policymaking, practice and advocacy. Members have expertise in political economy analysis, well-being in developing countries, social and public policy and international development.

The Advisory Group is engaged in an iterative process to refine the EGM framework by providing feedback on theories and methodologies, priority areas and the overall trajectory of the EGM. For example, the conceptual framework outlined in the following section has been informed by insights from the Advisory Group whilst ensuring that authorship remains clear and independent, without being diluted by potentially competing contributions.

To enhance the EGM framework, the Advisory Group will convene up to three times during the development and drafting of the EGM findings. Meetings will last approximately 1.5 hr and be conducted online via Teams to accommodate members across different regions and time zones. Between meetings, stakeholders can be invited to review draft documents and provide feedback via email. After each session, a summary of discussions, the meeting recording and key decisions will be shared to ensure transparency.

Membership remains flexible, allowing for the inclusion of additional stakeholders, particularly those from underrepresented regions or specialised fields.

### Conceptual Framework

This EGM is organised around two primary dimensions – intervention types and well-being outcomes – whose theoretical underpinnings are outlined below. The relationship between these dimensions reflects an underlying theory of change: that interventions to reduce or prevent the effects of climate change on populations vulnerable to poverty and social exclusion in LMICs can, in doing so, support or protect well-being across its material, relational and experiential dimensions.

More specifically, this EGM uses the term ‘well-being outcomes’ to describe the outcomes dimension of the evidence matrix. Well-being is increasingly understood in the literature as an inherently multidimensional concept that resists reduction to any single domain. Pinem et al. (2024) argue that well-being must be embraced as a holistic concept encompassing material conditions, subjective experiences, social relationships, cultural values and environmental sustainability, which emphasises the interdependence of various elements within a larger context and underlines the urgent necessity to comprehend well-being holistically, particularly for individuals and communities in the Global South. This understanding resonates across a diverse body of frameworks and traditions. Smith’s (2022) Ontology of Well-Being Thesis (TOWT) grounds well-being in six features of the human condition, namely embodiment, finiteness, sociability, cognition, evaluation and agency. Raworth’s (2017) Doughnut Economics similarly reframes well-being beyond material prosperity, situating human flourishing within both social foundations and planetary boundaries. Beyond Western traditions, non-Western frameworks articulate equally compelling and arguably more community-grounded conceptions of well-being. Ubuntu philosophy, rooted in the Bantu-speaking traditions of sub-Saharan Africa, holds that personal fulfilment is inseparable from communal harmony and collective welfare, captured in the maxim ‘a person is a person through other persons’ (Metz, 2012). Buen Vivir, emerging from Latin American indigenous thought, understands well-being not as individual prosperity but as a fulfilled life achievable only through deep relationships within community, extended to encompass other living beings and the environment (Gudynas, 2011). Both traditions challenge Western-centric assumptions about what well-being is and who defines it, which is a challenge directly relevant to an EGM centred on populations in the Global South.

Against this broader conceptual landscape, several established frameworks offer operational tools for measuring well-being across multiple dimensions, including the OECD’s well-being framework (OECD, 2024), McGregor et al. (2015) well-being approach, Gupte and te Lintelo (2015) and Menk et al. (2022). However, while each of these contributes valuable analytical tools, none is grounded in a consensual approach, derived from what people themselves identify as necessary for a decent life, to the extent that the Levitas et al.’s (2007) framework is. This is a significant consideration for an EGM focused on populations vulnerable to poverty and social exclusion in LMICs, where well-being is not only multidimensional but deeply situated in lived experience, collective circumstance and structural disadvantage. The consensual foundation of the framework by Levitas et al. (2007) aligns more closely with the community-centred and relational epistemologies reflected in Ubuntu and Buen Vivir, as well as with the holistic understanding advanced by Pinem et al. (2024) than frameworks developed primarily within or for high-income OECD settings.

This EGM therefore adopts a transdisciplinary approach to well-being outcomes, drawing on social policy, economics, sociology, psychology and anthropology to capture the full range of mechanisms through which climate change interventions may bring about change. Consistent with a human development lens (Sen, 1999) and in keeping with the theoretical framing that underpins this EGM, well-being outcomes are understood not through a deficit lens of poverty or exclusion but in terms of how interventions support or impinge upon people’s resources, participation and quality of life as the three primary dimensions of the Levitas et al.’s (2007) framework.

This framing aligns closely with Bronfenbrenner’s (1979) socio-ecological model, which conceptualises human development as shaped by nested and interacting layers of influence spanning the individual, household, community, institutional and global levels. The model is particularly apt here because it makes visible the multi-level pathways through which climate change affects well-being. As shown in Figure 1 above, its effects are simultaneously felt in the immediate environments of daily life such as family structures, kinship ties and access to local resources, and in the broader institutional, economic and political structures that govern people’s lives, often without their direct involvement. At the outermost level, overarching cultural values, global governance architectures and financial systems shape the conditions within which individuals and communities navigate climate-related risks. Critically, climate change operates as a transversal stressor across all these levels simultaneously, which is precisely why a framework capable of capturing multi-level interactions is necessary rather than merely useful.

Together, the Levitas et al.’s (2007) framework and Bronfenbrenner’s (1979) socio-ecological model provide complementary analytical lenses. The former specifies what dimensions of well-being this EGM tracks; the latter specifies at what level interventions are expected to operate. This combination is particularly well-suited to an EGM on climate change and social vulnerability, where the evidence base is heterogeneous, the populations affected are multiply disadvantaged and the mechanisms of change are rarely linear or confined to a single domain. A diagram illustrating these connections is provided below, with arrows indicating relationships between well-being dimensions and socio-ecological levels.

Lastly, recognising that the sources of knowledge underpinning interventions shape their design, implementation and contextual fit, this EGM will code the evidence base by knowledge type, including Indigenous and local knowledge, practitioner knowledge and knowledge derived from social research. This reflects the epistemological position that plural and place-based knowledge systems, including those rooted in community experience and non-Western traditions, constitute legitimate and analytically relevant sources of evidence, particularly in LMIC contexts where local knowledge may be central to intervention effectiveness.

### Dimensions

This EGM will gather evidence on different types of intervention that prevent or reduce the effects of climate change on populations vulnerable to poverty and social exclusion in LMICs. The EGM dimensions are illustrated in Table 1 above.

Studies will be coded against the ten domains of the Bristol Social Exclusion Matrix (Levitas et al., 2007), organised under three fields: resources, participation and quality of life.

Included reviews will be coded according to whether they incorporate, acknowledge or draw on Indigenous and local knowledge alongside research evidence in the design, implementation or evaluation of the intervention under review. Indigenous and local knowledge is understood here as the situated, experience-based knowledge held by Indigenous peoples and local communities about their environments, livelihoods and well-being, distinct from but not necessarily opposed to formalised research evidence (Berkes, 2008).

Where reviews are silent on knowledge sources, this will be coded as uncodeable and recorded transparently. The distribution of reviews across these categories will be reported as a substantive finding, given the growing policy emphasis on knowledge co-production in climate and development finance contexts.

The geographical scope filter will highlight where evidence is concentrated, distinguishing between rural and urban settings as well as climate-vulnerable regions. Country income classification will be recorded as a coding variable using the World Bank's (2025) sector taxonomy applicable at the time the systematic review was conducted, with historical classifications verified using the World Bank’s publicly available annual classification archives, where necessary.

The adoption of the 3ie taxonomy, together with the online 3ie taxonomy explorer, will provide precise definitions of studied interventions.

#### Types of Study Design

This EGM will include two types of systematic review: those assessing the effectiveness of eligible interventions and those assessing process evaluations or other studies of barriers and facilitators to highlight issues arising in the implementation of these interventions, as defined in the dimensions section above. Both completed and ongoing reviews will be eligible for inclusion to ensure the map stays relevant and reflects the latest available evidence.

The rationale for focusing on systematic reviews as the study design of interest is to offer a high-level, comprehensive, evidence-driven understanding of interventions from secondary studies. Systematic reviews encompassing qualitative, quantitative and mixed-methods approaches will be eligible across both types, since they enrich the map with evidence not only on the effects of interventions but on the mechanisms, barriers and facilitators that shape their implementation and outcomes. Qualitative systematic reviews are explicitly included where they meet the eligibility criteria below, particularly in the context of process evaluations, where qualitative methods are central to understanding implementation experiences and stakeholder perspectives.

For the purposes of this EGM, a systematic review is defined as a review meeting the following methodological criteria: a clearly stated set of objectives with pre-defined eligibility criteria; an explicit and reproducible search strategy; a systematic and transparent process for study selection and data extraction; and an assessment of the quality or risk of bias of included studies. The label ‘systematic review’ need not appear in the title, provided these criteria are met and documented.

Umbrella reviews (‘reviews of reviews’) that systematically synthesise the findings of existing systematic reviews will be included where they meet the above criteria and cover interventions and outcomes within the scope of this EGM. Where an umbrella review overlaps substantially with other included reviews, this will be recorded to avoid double-counting of evidence.

Scoping reviews will also be included where they employ a comprehensive and reproducible search strategy and address interventions or outcomes within the EGM scope. However, scoping reviews will not be assigned a confidence rating, given that quality appraisal of primary studies is not a standard feature of scoping review methodology. Their inclusion will be flagged transparently in the EGM report.

Other evidence and gap maps will be included where they employ a comprehensive and reproducible search strategy and address interventions or outcomes within the scope of this EGM. As no existing EGM is known to have mapped the specific intersection of climate change interventions, well-being outcomes and socially vulnerable populations in LMICs that this EGM addresses, any included EGMs will by definition cover adjacent thematic areas.

Systematic reviews that meet the eligibility criteria of this EGM but identify no primary studies will be included in the map. Such reviews, sometimes referred to as ‘empty reviews’, will be treated as substantive evidence of absence rather than absence of evidence. Empty reviews will be clearly flagged as such in the EGM report and will contribute to the identification of priority areas for future research.

Examples of eligible reviews are given below:

(1) Scheelbeek et al. (2021) conducted a systematic review of peer-reviewed literature reporting the effects on health of climate change adaptation responses in LMICs and identified 99 studies from 66 LMICs, reporting outcomes including reduction in infectious disease, improved access to water and sanitation and improved food security. This review reports well-being-relevant outcomes across multiple B-SEM domains. Although it does not explicitly target populations vulnerable to poverty and social exclusion, it notes – under ‘study populations’ – that findings also refer to low-income groups and children.
(2) Silchenko and Murray (2023) conducted a systematic literature review of 28 studies identifying evidence for how social protection has influenced beneficiaries’ migration decisions, experiences and outcomes in the context of climate change through cash transfers, public work programmes, insurance and healthcare.
(3) Greene et al. (2025) conducted a rapid umbrella review to synthesise recent evidence across systematic reviews of adaptation interventions and poverty in order to provide an evidence base to guide investment decisions, support scale-up of promising approaches and contribute to policy and practice in LMICs, including key enablers and barriers for successful adaptation and benefits for targeted social groups therein.

#### Types of Intervention/Problem

The eligibility criteria for interventions are defined by the PICO population shown in Table 2 below, drawing on White et al. (2020), in which the rows and columns of the EGM matrix correspond to the I and the O in the PICOs, supporting both the search strategy and the coding process.

**Table 2.** PICO Framework

Category descriptions	
Population (P)	Populations vulnerable to poverty and social exclusion (Levitas et al., 2007) in low- and middle-income countries (World Bank, 2024)
Intervention (I)	Interventions to reduce or prevent the effects of climate change on well-being
Comparison (C)	Lack of interventions or other interventions
Outcomes (O)	Well-being outcomes concerning resources, participation and quality of life
Study design (S)	Systematic reviews assessing the effectiveness (including the perceived effectiveness) of relevant interventions, as well as systematic reviews that examine process evaluations or other studies of implementation issues to explain how and why an intervention works

No restrictions are placed on the scale, duration or delivery mechanism of eligible interventions, in recognition of the heterogeneous and frequently complex nature of climate change interventions in LMIC contexts. Both single-component and multi-component interventions are eligible, whether these be at the individual, household, community, institutional or policy level, as outlined in the dimensions section above. Co-interventions will be recorded as a coding variable where reported. Eligible interventions include, but are not limited to, climate adaptation measures such as early warning systems, drought-resistant agricultural programmes and flood resilience infrastructure; social protection schemes such as cash transfers, food assistance and livelihood diversification programmes; health system strengthening interventions; community-based natural resource management initiatives; and policy and governance interventions targeting climate-vulnerable populations.

Where a systematic review addresses a scope broader than the PICOs, for example by including interventions beyond those covered by this map, or by examining populations that include both groups vulnerable to poverty and social exclusion and the general population, the following procedures will apply. Where data for eligible interventions or eligible populations are reported separately within the review, the authors will extract and include data solely from the eligible components. Where a review covers both socially vulnerable and broader populations but does not disaggregate findings by vulnerability status, the review will nonetheless be included and coded against the relevant Bristol SEM domains on the basis of available demographic, geographic and socio-economic descriptors, with the absence of disaggregated data recorded transparently as a coding limitation. Where a review’s scope is so broad that no meaningful extraction of eligible components is possible, it will be excluded and this decision documented.

In terms of additional eligibility criteria, only reviews published from 1990 onwards will be considered for inclusion, reflecting the period during which climate change began to be systematically addressed in the international policy and research literature. Reviews in any language will be included provided that titles and abstracts are available in English. Studies will be included regardless of publication type, encompassing academic journals, conference proceedings, books and book chapters, reports, theses and dissertations, grey literature and online publications.

Exclusion criteria:

• Primary research studies
• Reviews that do not assess interventions designed to reduce or prevent the effects of climate change
• Studies that are not systematic reviews of intervention effectiveness or process evaluations
• Reviews not reporting well-being outcomes across the domains of the Bristol B-SEM framework
• Reviews where neither title nor abstract is available in English
• Reviews in which all included primary studies were conducted exclusively in high-income countries.

#### Types of Population 

##### Inclusion

Included reviews will concern populations vulnerable to poverty and social exclusion in LMICs, at the level of individuals, households and/or communities. All age groups are eligible for inclusion. Vulnerability to poverty and social exclusion is operationalised through the Bristol Social Exclusion Matrix (Levitas et al., 2007), encompassing the domains of resources, participation and quality of life. For inclusion purposes, a population will be considered vulnerable where the review provides sufficient demographic, geographic or socio-economic descriptors to indicate exposure to disadvantage across one or more B-SEM domains, including but not limited to interventions addressing material deprivation, limited access to services, restricted social participation or adverse living conditions because of climate change.

##### Exclusion

Reviews will be excluded where all included primary studies were conducted exclusively in high-income countries or where the review population is demonstrably drawn from contexts of generalised prosperity with no discernible exposure to poverty or social exclusion as defined by the B-SEM framework.

#### Types of Outcome Measures 

For the purposes of eligibility, a review will be included if it reports at least one outcome related to human well-being, broadly defined. The governing eligibility framework for outcomes is the B-SEM framework (Levitas et al., 2007), organised under three fields – resources, participation and quality of life – which provide the primary coding scaffold for the EGM. No restrictions are placed on the measurement tools or instruments used to assess outcomes; self-reported, observational, administrative and participatory measures are all eligible, provided the outcome reported maps onto one or more B-SEM domains. Where a review reports outcomes across multiple domains, all relevant domains will be coded.

The ten domains of the B-SEM, as defined by Levitas et al. (2007), are as follows:

Resources

• Material and economic resources
• Access to public and private services
• Social resources

Participation

• Economic participation
• Social participation
• Culture, education and skills
• Political and civic participation

Quality of life

• Health and well-being
• Living environment
• Private safety

Reviews will be excluded where they report no outcomes mappable onto any B-SEM domain, or where reported outcomes relate exclusively to environmental or biophysical indicators with no discernible human well-being dimension.

#### Other Eligibility Criteria

##### Types of Location/Situation

Regarding the types of locations, this EGM will include studies conducted in urban, rural and peri-urban settings within LMICs. Settlement type will be recorded as a coding variable to surface any concentration or absence of evidence across different settings as a substantive finding.

##### Types of Settings

This EGM will consider interventions applied in a variety of environments, such as community-based settings, healthcare settings, educational institutions, urban planning initiatives and disaster-prone localities. These settings are relevant to the scope of this EGM, as interventions in each of these domains can have significant impacts on the well-being of socially vulnerable populations.

### Search Methods and Sources

For this EGM, bespoke search strings will be developed by conducting a thorough review and analysis of the topic under investigation. This will ensure that the search strategy is tailored to the specific focus of the EGM. The search queries will be designed using controlled vocabulary and/or free-text terms relevant to the key concepts and definitions outlined in Table 3 below.

**Table 3.** Key Concepts for Search Strategy

PICOs components	Search terms
Population: People vulnerable to poverty and social exclusion in LMICs	povert*; poor; “low-income”; “social exclusion”; “socially excluded”; marginali?ed; “socially vulnerable”; “vulnerable population*”; “at-risk population*”; “displaced person*”; “internally displaced”; refugee*; asylum*; “informal settlement*”; slum*; “urban poor”; “rural poor”; “food insecur*”; “food poverty”; “gender-based violence”; women; girl*; indigenous*; “traditional communit*”; “local communit*”; “ethnic minorit*”; “raciali?ed group*”; “low-income household*”; “subsistence farmer*”; “smallholder*”; “fragile state*”; “conflict-affected”; “post-conflict”; “low-income countr*”; “middle-income countr*”; LMIC*; “developing countr*”; “global south”; “least developed countr*”; “sub-Saharan Africa”; “South Asia”; “Southeast Asia”; “Latin America”; “Caribbean”; “Pacific Island*”; “socioeconomic disadvantage*”; “socioeconomic inequalit*”; “health inequalit*”; “health inequit*”
Intervention: Interventions to reduce or prevent the effects of climate change	”climate change”; “climate impact*”; “climate effect*”; “climate risk*”; “climate shock*”; “climate stress*”; “climate variabilit*”; “climate hazard*”; “climate adaptation”; “climate resilience”; “climate-resilient”; “adaptation intervention*”; “adaptation strateg*”; “adaptation measure*”; “adaptive capacit*”; “climate-smart”; “extreme weather”; “extreme heat”; drought*; flood*; heatwave*; “heat stress”; “sea level rise”; “storm surge”; cyclone*; hurricane*; typhoon*; desertification; “land degradation”; “soil degradation”; “erratic rainfall”; “rainfall variabilit*”; “climate-related disaster*”; “natural disaster*”; “disaster risk reduction”; “disaster risk management”; “early warning system*”; “social protection”; “cash transfer*”; “safety net*”; “livelihood*”; “livelihood diversification”; “community-based adaptation”; “nature-based solution*”; “ecosystem-based adaptation”; “water securit*”; “water management”; WASH; “food system*”; “agricultural adaptation”; “climate-smart agriculture*”; “health system*”; “health intervention*”; “displacement”; “migration”; “planned relocation”
Study design: Systematic evidence syntheses (effectiveness and process evaluations)	“systematic review*”; “systematic literature review*”; “review of reviews”; “umbrella review*”; “overview of reviews”; “scoping review*”; “scoping stud*”; “meta-analysis”; “meta-analys*”; “meta-synthesis”; “meta-ethnograph*”; “evidence synthesis”; “evidence and gap map*”; “evidence gap map*”; “rapid review*”; “rapid evidence assessment*”; “Cochrane review*”; “Campbell review*”; “what works”; “realist review*”; “realist synthesis”; “narrative review*”; “integrative review*”; “process evaluation*”; “implementation review*”

In order to maximise the comprehensiveness of the search, a detailed list of search terms will be created from existing studies and stakeholder interests, incorporating synonyms, wildcards and thesauri where appropriate. This will help ensure that the search captures all relevant studies, including those using different terminology or phrasing. The search will cover multiple bibliographic databases to guarantee a broad and inclusive collection of evidence.

The search will be conducted during the period between May and June 2026. In order to create an effective search strategy for this EGM, the following concepts will be combined using Boolean operators. Table 3 below shows the key concepts that could be adopted to develop the search strings.

The concepts outlined in Table 3 above have undergone a preliminary pilot search to evaluate their relevance and capacity to identify pertinent keywords for use as free-text terms, as well as to recognise controlled vocabulary. An iterative and purposive approach to searching (Patton, 1990) will be adopted, continuing until thematic saturation is deemed to have been achieved (Bowen, 2008; O’Reilly & Parker, 2013). However, it is acknowledged that the concept of data saturation has limitations, as new data may emerge, introducing novel themes that offer fresh exploratory insights (Wray et al., 2007). To mitigate this issue, searches will be re-run prior to the final analysis, with any additional studies identified being retrieved for inclusion.

What follows is a sample search string for Web of Science (Core Collection) combining all concepts:

**Box 1**

povert* OR poor OR “low-income” OR “social exclusion” OR “socially excluded” OR marginali?ed OR “socially vulnerable” OR “vulnerable population” OR “vulnerable populations” OR “at-risk population” OR “at-risk populations” OR “displaced person” OR “displaced persons” OR “internally displaced” OR refugee* OR asylum* OR “informal settlement” OR “informal settlements” OR slum* OR “urban poor” OR “rural poor” OR “food insecurity” OR “food insecure” OR “food poverty” OR “gender-based violence” OR indigenous* OR “traditional community” OR “traditional communities” OR “local community” OR “local communities” OR “ethnic minority” OR “ethnic minorities” OR “low-income household” OR “low-income households” OR “subsistence farmer” OR “subsistence farmers” OR smallholder* OR “fragile state” OR “fragile states” OR “conflict-affected” OR “post-conflict” OR “low-income country” OR “low-income countries” OR “middle-income country” OR “middle-income countries” OR LMIC* OR “developing country” OR “developing countries” OR “global south” OR “least developed country” OR “least developed countries” OR “sub-Saharan Africa” OR “South Asia” OR “Southeast Asia” OR “Latin America” OR “Caribbean” OR “Pacific Island” OR “Pacific Islands” OR “socioeconomic disadvantage” OR “health inequalities” OR “health inequity” OR “health inequities”

AND

“climate change” OR “climate impacts” OR “climate effects” OR “climate risk” OR “climate risks” OR “climate shock” OR “climate shocks” OR “climate stress” OR “climate variability” OR “climate hazard” OR “climate hazards” OR “climate adaptation” OR “climate resilience” OR “climate-resilient” OR “adaptation intervention” OR “adaptation interventions” OR “adaptation strategy” OR “adaptation strategies” OR “adaptation measure” OR “adaptation measures” OR “adaptive capacity” OR “climate-smart” OR “extreme weather” OR “extreme heat” OR drought* OR flood* OR heatwave* OR “heat stress” OR “sea level rise” OR “storm surge” OR cyclone* OR hurricane* OR typhoon* OR desertification OR “land degradation” OR “soil degradation” OR “erratic rainfall” OR “rainfall variability” OR “climate-related disaster” OR “climate-related disasters” OR “natural disaster” OR “natural disasters” OR “disaster risk reduction” OR “disaster risk management” OR “early warning system” OR “early warning systems” OR “social protection” OR “cash transfer” OR “cash transfers” OR “safety net” OR “safety nets” OR livelihood* OR “livelihood diversification” OR “community-based adaptation” OR “nature-based solution” OR “nature-based solutions” OR “ecosystem-based adaptation” OR “water security” OR “water management” OR WASH OR “food system” OR “food systems” OR “agricultural adaptation” OR “climate-smart agriculture” OR “health system” OR “health systems” OR “health intervention” OR “health interventions” OR displacement OR migration OR “planned relocation”

AND

“systematic review” OR “systematic reviews” OR “systematic literature review” OR “review of reviews” OR “umbrella review” OR “umbrella reviews” OR “overview of reviews” OR “scoping review” OR “scoping reviews” OR “scoping study” OR “scoping studies” OR meta-analysis OR meta-analyses OR “meta-synthesis” OR “meta-ethnography” OR “evidence synthesis” OR “evidence and gap map” OR “evidence gap map” OR “rapid review” OR “rapid reviews” OR “rapid evidence assessment” OR “Cochrane review” OR “Campbell review” OR “what works” OR “realist review” OR “realist synthesis” OR “narrative review” OR “integrative review” OR “process evaluation” OR “process evaluations” OR “implementation review”

The query link used is: https://www.webofscience.com/wos/woscc/summary/dd00d04a-e57d-422b-bef7-b98a8d3d8b2a-01b238b069/relevance/1.

This has yielded 9,606 results, including Scheelbeek et al. (2021) and Silchenko and Murray (2023), presented above as examples of reviews likely to meet the eligibility criteria of this EGM. Greene et al. (2025), also cited above as an example, was identified as grey literature through a search of the FCDO Research and Evidence repository.

It is anticipated that various terms, particularly those primarily associated with health, will have been utilised in primary studies and then captured in evidence syntheses to describe the multifaceted and interconnected effects of climate change on human well-being. However, the definition of well-being adopted in this review is intentionally broader, encompassing not only health but also the social and economic dimensions as well as the cultural and political aspects that collectively contribute to enhancing the quality of life for the populations under study. This potential semantic challenge will be addressed by incorporating terms from a broad range of literatures, including, but not limited to, public health, psychology, sociology, economics and anthropology. By doing so, the search terms will capture the full breadth of conceptualisations of well-being, as articulated across these diverse academic fields.

Lastly, scoping reviews will be included in this EGM as they provide a systematic overview of broad and often complex areas where evidence may be diverse and uneven. While they differ from traditional systematic reviews in focus and synthesis, the authors maintain that these can still offer valuable insight into where research is concentrated and where significant gaps remain.

#### List of Proposed Search Databases

Sources include (but are not limited to) bibliographic databases, reference lists of included studies, key journals, conference proceedings, Internet resources and contact with study investigators, experts and humanitarian and development practitioners.

Examples of electronic bibliographic databases include:

• ERIC (Education Resources Information Centre) (ProQuest)
• Medline (ProQuest)
• PsycINFO (EBSCO)
• Web of Science (Web of Knowledge)
• ASSIA (Applied Social Sciences Index and Abstracts) (ProQuest)
• Social Services Abstracts (ProQuest)
• IBSS (International Bibliography of the Social Sciences) (ProQuest)
• Sociological Abstracts (ProQuest)
• Econlit (EBSCO)
• Scopus

#### Grey Literature and Supplementary Sources

• Websites: Relevant websites of research institutions and development agencies (e.g., 3ie Development Evidence Portal, Campbell Collaboration Library, FCDO Research and Evidence, Overseas Development Institute, World Bank Open Knowledge Repository, FCDO Research, IMF eLibrary, ReliefWeb).
• Backward and forward citation tracking
• Personal contacts: Area specialists will be contacted for information regarding potentially relevant studies. For non-English studies, official translations will be requested. If unavailable, titles and abstracts will be translated into English using DeepL.
• Conference proceedings, dissertations and theses.
• Networks: Requests for relevant studies will be made by contacting members of pertinent networks, such as:
  ◦ Caritas Internationalis https://www.caritas.org
  ◦ The Sustainable Welfare and Eco-social Policy Network, University of Hamburg https://www.wiso.uni-hamburg.de/en/fachbereich-sozoek/professuren/zimmermann/ecowelfarenet.html
  ◦ The International Institute for Environment and Development https://www.iied.org
  ◦ The Loss and Damage Collaboration https://www.lossanddamagecollaboration.org

### Analysis and Presentation

#### Report Structure

The EGM report will be structured in three main parts. The first will provide the background, conceptual framework and objectives of the EGM, including a discussion of existing evidence and the rationale for the map. The second will set out the methods in full, covering eligibility criteria, search strategy, data extraction, coding, critical appraisal and stakeholder engagement. The third will present the findings, including the visual EGM, a narrative analysis of evidence characteristics and trends, and a discussion of key gaps and priorities for future research.

#### Filters for Presentation

For the purposes of this EGM, additional dimensions have been identified that are relevant to stakeholders and that can enhance the understanding of how interventions are related to well-being outcomes in different contexts. The following filters will guide and inform the development of the EGM:

• Country: The reviews must be about or conducted in low- and middle-income countries (LMICs), as defined by the World Bank’s (2024) income classification.
• Delivery channel or place: The setting in which interventions are delivered will be categorised into:
  ◦ Micro- and meso-system level interventions, such as interventions provided in the household or domestic environment; interventions in educational settings, including pre-school, primary and secondary schools; interventions provided in clinics, hospitals or other formal health settings; interventions implemented at the community level, including local administration.
  ◦ Macro-system (cultural and societal) level interventions, such as implementation of the Paris Agreement or national climate action plans to reduce carbon emissions; debt relief initiatives for climate-vulnerable nations to enhance resilience; environmental laws, carbon taxes and climate litigation cases influencing global or national policies; large-scale public awareness campaigns promoting sustainable consumption (e.g., reducing reliance on fossil fuels).
  ◦ Exo-system (institutional and structural) level interventions, such as IMF and World Bank climate finance initiatives supporting adaptation projects in low-income countries; funding for climate resilience projects by organisations like the Green Climate Fund (GCF) or UNDP; large businesses adopting ESG (Environmental, Social and Governance) principles to reduce environmental harm; policies ensuring fair trade and climate-resilient agricultural (agro-ecological) practices.
• Gender: Reviews will be coded according to whether they explicitly assess outcomes disaggregated by gender or focus specifically on a gendered population. Reviews that include participants of all genders without disaggregation will be coded as not gender-disaggregated; this will be recorded as a coding characteristic rather than grounds for exclusion.
• Disability: Reviews will be coded according to whether they explicitly focus on populations with disabilities, whether these be physical, mental or both, or explicitly disaggregate outcomes by disability status. Reviews that include participants with disabilities within a broader mixed population without disaggregation will be coded accordingly, with the absence of disaggregation recorded transparently.
• Socio-economic Status (SES): Studies will be filtered based on the socio-economic status of the population being studied. This can include factors such as income level, education level, and occupation.
• Employment and occupation: Vulnerabilities related to employment status, job type or occupational safety will be considered. Categories may include unemployed or underemployed; informal sector workers; vulnerable occupations (e.g., agricultural workers, domestic workers).
• Living Conditions: This filter will focus on individuals living in poor or substandard housing conditions, such as slums or informal settlements. Categories may include informal housing (e.g., slums, crowded urban settlements) or poor housing quality (e.g., inadequate access to basic utilities like electricity, water, sanitation).
• Discrimination and marginalisation faced by populations due to ethnicity, caste, religion, or other factors. Categories may include ethnic minorities, refugees or displaced persons, marginalised or excluded social groups.
• Vulnerabilities linked to pre-existing conditions (Stanger, 2011): This filter will capture populations whose vulnerability is linked to specific health conditions, such as chronic illness, mental health conditions, or physical disabilities.

#### Dependency

The unit of analysis is individual systematic reviews. Where a review has been published in more than one outlet – for example in both ERIC and a subject journal – these will be grouped into a single entry, with the most complete or most recently updated version designated as the primary record, to prevent double counting of evidence.

### Data Collection and Analysis

#### Screening and Study Selection

The selection of systematic reviews will adhere to the pre-established eligibility criteria outlined above. The screening process for the EGM will be conducted systematically and collaboratively to ensure rigour and consistency. Alfonso Apicella (AA), as the lead reviewer, will oversee the initial development of the search strategy, conduct the primary screening of titles and abstracts, and undertake the full-text review to determine study eligibility based on the predefined inclusion and exclusion criteria. Sandy Oliver (SO) and Janice Tripney (JT) will independently review distinct subsets of studies to ensure consistency and accuracy in the screening process.

In order to maintain methodological integrity, the reviewers will engage in regular discussions to resolve any discrepancies and ensure adherence to the established criteria. Additionally, the potential involvement of subject-matter experts and master’s students may be considered to provide further perspectives and enhance the robustness of the review. Any disagreements arising during the screening process will be resolved through consensus.

The criteria will be piloted by the lead reviewer who will screen studies on titles and abstracts.

##### Phase One: Title and Abstract Screening

In the initial phase, all three authors will be involved in piloting the selection criteria and the title/abstract screening phase. Subsequently, AA will start screening the titles and abstracts of records identified via electronic database searches once there is agreement amongst the authors. Each item’s relevance will be evaluated through a single screening process, with decisions documented using EPPI-Reviewer software. Items will be included unless they clearly fail to meet the criteria based on title and abstract information.

Any uncertainty regarding eligibility will be noted as ‘unsure’ and discussed among co-authors. Non-English reviews with English titles and/or abstracts will be translated using DeepL for screening against the selection criteria. Manual searches will be conducted concurrently with electronic searches and eligibility determined by AA, maintaining electronic records of eligible items and those causing doubt, following similar procedures for non-English or title-only items.

##### Phase Two: Full-Text Screening

Full-length reports of studies passing the initial screening or marked as ‘unsure’ will be obtained. AA, SO and JT will independently examine different subsets of full-length reports. Any discrepancies will be resolved by identifying the source of disagreement, re-reading and discussion. If consensus cannot be reached, the involvement of additional reviewers will be sought from within the UCL EPPI Centre. All study selection and information retrieval activities will be thoroughly documented in the final report for replicability and clarity, accompanied by summary flowcharts.

#### Data Extraction and Management

For data extraction, information about study design and methodology, participant demographics, stated vulnerabilities and climate change impacts will be collected. Further information will be sought from study investigators for unreported data or additional details, when necessary.

Piloting of the coding tool will be conducted by all three authors, who will work independently on a purposeful sample of eligible studies (chosen to test the tool across all included study designs) before comparing decisions. Any coding discrepancies will entail reworking specific items, leading to adjustments in the coding book.

This process will continue until a high level of consistency in code application is achieved. The remaining studies will be coded by AA who will independently extract information from each study report.

Data from the included studies will be extracted using a structured and pre-tested coding tool to ensure consistency and accuracy. AA, SO and JT will extract data independently to minimise errors and reduce bias.

Automation and machine learning will be considered for data extraction, organisation of preliminary data and detection of duplicates. Any use of AI tools will be reported in full accordance with the RAISE guidelines for AI-assisted systematic reviews, as endorsed by Campbell (Flemyng et al., 2025), including specification of which tools were used, how they were applied and at what stage of the process. Data extraction will be carried out by AA.

The following key information will be extracted from each included study:

(1) Study characteristics
  ◦ Citation details (authors, year, title, journal, DOI)
  ◦ Study design (systematic review, meta-analysis)
  ◦ Geographic location (country, region, urban/rural classification)
  ◦ Population characteristics (age, gender, socio-economic status, disability status, etc.)
  ◦ Sample size and key demographics
(2) Intervention characteristics will be mapped against the levels of the social ecological model (SEM), with further refinement using the 3ie taxonomy for more detailed classification. The following features will be captured, when available:
  ◦ Type of intervention, such as policy interventions (e.g., climate policy frameworks, adaptation strategies), social protection programmes (e.g., cash transfers, health insurance schemes) or climate-specific measures (e.g., mitigation efforts, infrastructure resilience building).
  ◦ Financing mechanism: Where reported, the primary financing source of the intervention will be coded under the following categories: multilateral (e.g., World Bank, IMF, IFAD, regional development banks); bilateral (e.g., USAID, FCDO); blended finance (combining public and private capital); domestic public spending; NGO or philanthropic funding; and community-led or informal financing. Where the financing source is not reported, this will be recorded as uncodeable. The distribution of reviews across financing categories will be reported as a substantive finding, given the significant role of development finance in shaping what interventions are implemented and for whom.
  ◦ Intervention components and delivery mechanisms, such as cash transfer schemes, educational outreach programmes, public awareness campaigns, infrastructure upgrades and community-based initiatives. The delivery mechanism may also take into account whether the intervention is top-down (e.g., government-led policies) or bottom-up (e.g., grassroots mobilisation, community-led efforts).
  ◦ Stakeholder involvement and knowledge co-production: Reviews will be coded according to whether they address mechanisms of stakeholder involvement in the design, implementation or evaluation of the intervention, including participatory processes, community engagement, knowledge translation practices, evidence portals or co-production arrangements. This coding dimension will identify where the evidence base addresses the conditions under which interventions are developed *with* rather than *for* affected populations. This helps generate findings directly relevant to understanding evidence-informed decision-making in development contexts.
  ◦ Duration and frequency: where a systematic review reports on the temporal characteristics of included interventions, such as whether evidence is concentrated in short-term emergency responses or long-term sustainability programmes, this will be recorded descriptively as a characteristic of the review’s evidence base.
  ◦ Target population will be coded against the ten domains of the Bristol Social Exclusion Matrix (Levitas et al., 2007) to enable systematic identification of which dimensions of social vulnerability are addressed by included interventions. Where reviews do not explicitly characterise their target populations in vulnerability terms, coding will draw on available demographic, geographic and socio-economic descriptors and use those to infer which B-SEM domains are relevant. Where insufficient information is available to code a domain with confidence, this will be recorded as uncodeable rather than inferred, ensuring coding decisions remain transparent and replicable
  ◦ Effectiveness: Reviews will be coded according to their overall effectiveness conclusion. Information on unintended outcomes and barriers to successful implementation, such as political, economic or logistical constraints, will be extracted and reported descriptively, where available. All effectiveness coding will be based on the systematic review’s own conclusions rather than independent appraisal of primary study findings.
  ◦ Political economy context: Where a systematic review addresses the political economy conditions under which an intervention was designed or implemented, including stakeholder power dynamics, institutional incentives, implementation barriers of a political or economic nature, or the conditions under which evidence was or was not taken up by decision-makers, this will be recorded descriptively. This dimension is not a screening criterion but a coding variable and is intended to surface patterns in the evidence base that can help understand how certain interventions are framed, shaped and implemented.
(3) Outcomes of interest
  ◦ Well-being outcomes assessed by the systematic review, coded against the outcome domains of the EGM framework
  ◦ Where a systematic review reports a range of measurement tools or scales used across its included studies, this range will be noted descriptively rather than extracted at study level
  ◦ Any unintended or adverse outcomes reported by the systematic review, recorded descriptively
(4) Methodological quality and risk of bias
  ◦ The methodological quality of included systematic reviews will be assessed using AMSTAR 2 (Shea et al., 2017).
  ◦ Where a systematic review itself reports a risk of bias assessment of its included primary studies, the overall conclusion of that assessment will be recorded descriptively, along with a note of the range of risk of bias findings reported across included studies.
  ◦ Strengths and limitations as reported by the systematic review authors will be recorded descriptively.

If critical information is missing or unclear in a systematic review report, attempts will be made to contact the review authors via email or institutional websites, with a follow-up reminder sent if no response is received within four weeks.

#### Tools for Assessing Risk of Bias/Study Quality of Included Reviews

In order to ensure the robustness and reliability of included systematic reviews and meta-analyses, this EGM will employ AMSTAR-2 (Shea et al., 2017) as the primary tool for assessing methodological quality. This decision is informed by Perry et al. (2021), who found that while ROBIS is more effective for assessing risk of bias, AMSTAR-2 provides a more comprehensive evaluation of overall quality. Given the review team’s prior experience in applying AMSTAR-2, it will be adopted as the preferred tool for this EGM.

AMSTAR-2 will be applied systematically to all included systematic reviews by three independent reviewers. Systematic reviews will be categorised based on AMSTAR-2 ratings as high, moderate, low, or critically low in methodological quality. Discrepancies in assessments will be resolved through discussion, with a fourth reviewer consulted if necessary.

#### Methods for Mapping

EPPI-Reviewer 6, the current version of the software (Thomas et al., 2023), will be used to manage the review process. It supports collaboration across different locations and incorporates machine-learning tools to assist with screening and analysis.

Studies identified through electronic database searches will be imported into EPPI-Reviewer 6 and screened against the eligibility criteria. Any relevant studies found through non-electronic searches will be added manually to ensure comprehensive coverage of the evidence base.

## Supplemental Material

Supplemental Material - PROTOCOL: Interventions to Reduce or Prevent the Effects of Climate Change on Populations Vulnerable to Poverty and Social Exclusion in Low- and Middle-Income Countries: An Evidence and Gap Map Supplemental Material for PROTOCOL: Interventions to Reduce or Prevent the Effects of Climate Change on Populations Vulnerable to Poverty and Social Exclusion in Low- and Middle-Income Countries: An Evidence and Gap Map by Alfonso Apicella, Sandy Oliver, and Janice Tripney in Campbell Systematic Reviews.
