# Supplementary material for: PROTOCOL: Interventions to Reduce or Prevent the Effects of Climate Change on Populations Vulnerable to Poverty and Social Exclusion in Low- and Middle-Income Countries: An Evidence and Gap Map
Source: Campbell Syst Rev. 2026 Jul 21;22(3):18911803261461576. doi: 10.1177/18911803261461576 (PMC13518301; doi:10.1177/18911803261461576)
Supplement: Supplementary file 1 [file sj-pdf-1-cam-10.1177_18911803261461576.pdf]

Appendix A

Advisory Group members (in alphabetical order)

Name	Role / capacity	Web profile	Country
Allister McGregor	Academic	https://sustainable-care.sites.sheffield.ac.uk/people/team/allister-mcgregor	UK
Karina Padilla	International consultant	https://www.devsca.org/karina-padilla	Peru
Laura Camfield	Academic	https://www.kcl.ac.uk/people/laura-camfield	UK
Patrick Grady	Policymaker	https://patrickgrady.scot/	UK
Zan Akologo†	NGO practitioner	https://integralvoices.wordpress.com/samuel-zan-akologo/	Ghana

† Deceased in January 2025.

Appendix B

Terms of Reference for the Advisory Group (AG)

Why an Advisory Group?

My Social Science PhD programme started in January 2024 under the supervision of Professor Sandy Oliver and Dr Janice Tripney from the UCL Social Research Institute. Within our team, we believe that research, especially when policy-relevant, can be improved by involving key stakeholders whose voice permits a much more nuanced understanding of the issue under investigation, as well as its interpretation and application to real-world issues.

For this reason, an Advisory Group (AG) made up of volunteer academics, policymakers and practitioners is set up to allow for cross-fertilisation of ideas across a range of different disciplines, types of expertise and lived

experiences. This is believed to ultimately contribute to advancing research on the repercussions of climate change on human well-being in low- and middle-income countries, alongside their implications for social and public policy.

About the role

The AG is made up of volunteers from different disciplines and perspectives. It will offer a space to share experiences and insights that will ultimately inform the trajectory of my doctoral research. It is hoped that, as a space for sharing expert advice, it will provide members with a positive sharing experience – whoever they are, wherever they are based and whatever they role, from the moment they choose to offer their time and talents until they step down from their advisory role.

More specifically, the role of the AG is:

- Primarily to advise the doctoral researcher on the scope of the proposed study to ensure it is policy-relevant, fit-for-purpose, of high quality and addresses the needs of potential readers and end users.
- To provide insights grounded in diverse experiences, encompassing both the subject matter and the methodology used. This diversity of ‘voices’ will ensure that research decisions are made with the people whose lived experience is investigated at the heart of them.

What will I be doing?

Members will be asked to:

- Reflect and draw on personal academic/policy/practitioner experience and bring their ideas and expertise to contribute to discussions concerning the PhD topic to hand.
- Give feedback to the doctoral researcher on work as it progresses, such as draft processes, initial analyses, research claims, or the development of methodology.

What is the time commitment?

I aim to convene the AG two to three times a year, with meetings taking place online via Teams/Zoom and lasting about one hour and a half. Between

meetings there might be occasional requests to provide feedback on draft documents or read emails with progress updates.

If you can’t make a meeting, please give as much notice as possible. A recording of the meeting and summary of discussion points will be circulated after each meeting. Your opinion matters so I will hope to run meetings at a time that’s suitable to everyone.

This AG is launched in 2024 with the members who have generously agreed to take part in the research scoping process. New members might be added along the way, especially from underrepresented regions or areas of expertise, in order to enrich discussions.

Participation in the activities of the AG is voluntary and members can withdraw at any point. Likewise, new members can be added at any time, based on the evolving needs of the research project and in consultation with the existing group.

Categories of AG members sought

Participants falling into any of the following categories will be invited:

- Area experts, such as academics, independent researchers, etc.
- Policymakers, decision-makers and institutional figures
- NGO practitioners from e.g. social development agencies, humanitarian organisations, etc.

I will aim for gender balance and geographical diversity throughout.

Coordination responsibility

The postgraduate researcher will assume primary responsibility for coordinating the AG and establishing an effective communication mechanism. All communication should be initiated via the postgraduate researcher, who will ensure that all members are apprised.

Communication channels will reflect the available resources and preferences of the AG members, such as Teams/Zoom for meeting online at mutually convenient times from different time zones.

What will AG members get out of it?

- This is an opportunity to directly influence and mould the focus of my doctoral research in an area of interest adjacent to yours.
- Be part of a diverse team made up of experts interested in well-being and sustainability.
- Discuss ways of mobilising knowledge around the research topic at hand.

This document will be subject to periodic review and adjustments as needed during the research process.

Contact details

Alfonso Apicella, MSc
Postgraduate Researcher
UCL Social Research Institute, EPPI Centre
alfonso.apicella.18@ucl.ac.uk
